# Variation in Population Vulnerability to Heat Wave in Western Australia

**DOI:** 10.3389/fpubh.2017.00064

**Published:** 2017-04-03

**Authors:** Jianguo Xiao, Tony Spicer, Le Jian, Grace Yajuan Yun, Changying Shao, John Nairn, Robert J. B. Fawcett, Andrew Robertson, Tarun Stephen Weeramanthri

**Affiliations:** ^1^Public Health Division, Department of Health, Government of Western Australia, Perth, WA, Australia; ^2^School of Public Health, Curtin University, Perth, WA, Australia; ^3^Australian Bureau of Meteorology, Adelaide, SA, Australia; ^4^Australian Bureau of Meteorology, Melbourne, VIC, Australia

**Keywords:** heat wave, vulnerability, socioeconomic status, geographical variation, morbidity, mortality, Western Australia

## Abstract

Heat waves (HWs) have killed more people in Australia than all other natural hazards combined. Climate change is expected to increase the frequency, duration, and intensity of HWs and leads to a doubling of heat-related deaths over the next 40 years. Despite being a significant public health issue, HWs do not attract the same level of attention from researchers, policy makers, and emergency management agencies compared to other natural hazards. The purpose of the study was to identify risk factors that might lead to population vulnerability to HW in Western Australia (WA). HW vulnerability and resilience among the population of the state of WA were investigated by using time series analysis. The health impacts of HWs were assessed by comparing the associations between hospital emergency department (ED) presentations, hospital admissions and mortality data, and intensities of HW. Risk factors including age, gender, socioeconomic status (SES), remoteness, and geographical locations were examined to determine whether certain population groups were more at risk of adverse health impacts due to extreme heat. We found that hospital admissions due to heat-related conditions and kidney diseases, and overall ED attendances, were sensitive indicators of HW. Children aged 14 years or less and those aged 60 years or over were identified as the most vulnerable populations to HWs as shown in ED attendance data. Females had more ED attendances and hospital admissions due to kidney diseases; while males had more heat-related hospital admissions than females. There were significant dose–response relationships between HW intensity and SES, remoteness, and health service usage. The more disadvantaged and remotely located the population, the higher the health service usage during HWs. Our study also found that some population groups and locations were resilient to extreme heat. We produced a mapping tool, which indicated geographic areas throughout WA with various vulnerability and resilience levels to HW. The findings from this study will allow local government, community service organizations, and agencies in health, housing, and education to better identify and understand the degree of vulnerability to HW throughout the state, better target preparatory strategies, and allocate limited resources to those most in need.

## Introduction

A heat wave (HW) is a prolonged period of excessively hot weather. Heat waves have caused more deaths in Australia since European settlement than all other natural hazards combined, and are predicted to increase in frequency, duration, and intensity, with a doubling of the number of HW-related deaths in the next 40 years (1, 2). Currently, there is no standardized definition for HW internationally or among different jurisdictions in Australia. A recent study conducted in Western Australia (WA) found that the excess heat factor (EHF) metric was the best HW indicator among the three metrics examined to predict greatest health service demand (3). That study’s outcomes were only based on Perth’s metropolitan population, which required new studies to test the validity of EHF for the whole of WA.

Heat waves typically affect large geographical areas over the course of three or more days. Many jurisdictions, including WA, have created extreme heat emergency management plans to respond to HW events. With large populations and limited resources, many jurisdictions lack the precision to target the most at risk populations with appropriate public health interventions, and many HW plans are based on assumptions and research from other states and countries. There has been little verification of whether a particular population group is at higher risk or even resilient to HWs, although acclimatization, individual susceptibility, and community and geographical characteristics all affect heat-related effects on mortality and morbidity (2, 4–7). Past epidemiological studies have established consistently identifiable vulnerable groups to extreme heat. Young children and the elderly are commonly identified at high risk of morbidity and mortality during the period of HWs (8–11), whereas people with renal, respiratory, and cardiovascular conditions are susceptible to heat due to hyperthermia and dehydration (12–14). However, there have only been a limited number of studies examining the geographical variation and effects of socioeconomic status (SES) on people’s response to HW.

Western Australia is the largest state in Australia with varying geographic features and climates that range from temperate areas in the south to tropical areas in the north. Seventy-eight percent of the population is based in the Perth metropolitan area with the remaining 22% scattered throughout regional and remote areas. To improve preparedness and response arrangements for HWs, there is a need to determine which populations are at higher risk of heat exposure and what are the risk factors related to it. Our study aims to characterize the relationship between HW intensity and health service demand of different population groups in different regions of WA. By identifying vulnerable populations, agencies, service providers, and local government authorities can better target their limited resources to those populations most at risk.

## Materials and Methods

Western Australia is Australia’s largest state with an area of more than 2,500,000 km^2^ and over 12,500 km of coastline. It has a population of approximately 2.6 million people (15). The southwest corner of the state has a mediterranean climate (i.e., hot dry summers and cooler wet winters) where about 85% of the WA population lives. The central four-fifths of the State are semiarid or desert and are lightly inhabited. An exception to this is the northern tropical region that has an extremely hot monsoonal climate.

### Derive HW Intensity, Adjust for Delayed Effects of HW, and Identify Significant Health Service Usage Measures

Heat waves were measured using HW intensity at each geographical area represented by statistical local area (SLA) in WA. A HW day, defined by an EHF, was defined as the exceedance of the previous 3-day mean daily temperature (DT) above the 95th percentile threshold, multiplied by the difference between the 3-day mean DT and the mean of the prior 30 days. Nairn and Fawcett (16) provide the full equation. The EHF was then normalized and expressed as a heat wave severity index (HWSI), dividing the EHF by the long-term 85th percentile of positive EHF at every location. The HWSI data at SLA level were sourced from the Australian Bureau of Meteorology (BoM). The BoM identifies severe HWs when HWSI is greater than 1, which becomes extreme when HWSI is greater than 3. In our analysis, severe and extreme HW days were combined to severe/extreme HW days, as the counts for extreme HW days were very small and not suitable for a separate statistical analysis. Low-intensity HW days occur if the HWSI value was between 0 and 1; and non-heat wave days were defined as having a HWSI less than or equal to 0. The EHF was calculated over a 3-day period (16), and we applied the EHF value to the first day in an attempt to identify any possible delayed effects of HW.

Time series design was used for the study. The daily health service usage data from 1 November 2006 to 30 April 2015 for warm months (November to April in the following year) for the whole of WA was obtained and measured from following three datasets: (1) hospital admission data from WA hospital morbidity data system (HMDS), including overall (all admissions), cardiovascular diseases (defined as having a principal diagnosis of ICD-10-AM seventh Edition codes between I00-I99 plus G45), respiratory diseases (J00–J99), kidney diseases (N10–N19), and heat-related diseases (having a principal or any additional diagnosis of L55, L74.0, T67, X30, or X32); (2) overall emergency department (ED) attendance data from WA ED data collection; and (3) death data from WA registry of births, deaths and marriages. The chosen HMDS conditions were based on existing literature where conditions were identified as having a strong association with HWs (10, 17). The health service utilization rates in different HW intensities were compared with those during non-HW days for all aforementioned conditions.

Estimated resident populations (ERPs) by age group, gender, and SLA were sourced from Australian Bureau of Statistics. The monthly populations were computed using a linear interpolation method, based on mid-year ERPs. Such populations were then applied to all the days in the month of the study period. Daily health service usage rates were calculated by age group, gender, and SLA. The total population covered in the study period (i.e., warm months) from 1 November 2006 to 30 April 2015 was 11,698,702 person-years. To assess for the delayed effects of HW on health service usage, service usage rates were first derived by dividing daily service usage counts by daily populations on the same day as the HW day, or 2- to 30-day cumulative counts divided by corresponding cumulative populations.

A Pearson correlation analysis was then conducted between the EHF for a day (i.e., first day of the 3-day period) and its corresponding health service usage rate of that day. The rates or cumulative rates with the highest positive correlation with a significance value of 0.05 or less were then selected and used in the bivariate and multivariate statistical analyses to assess the potential risk factors of high service usage during HW exposure. Only health service usage measures with significant correlation with EHF were included in the further analyses below.

Sensitivity of datasets to HW was examined, and only results identified as having significant association with EHF will be reported in this paper.

### Determine Risk Factors and Interactions between Risk Factors and HW Intensity

A literature review was conducted to identify potential risk factors of HW. Age, gender, HW intensity, SES [measured by the socioeconomic index for areas (SEIFA)], and service accessibility [measured by the accessibility/remoteness index of Australia (ARIA)] were identified as key risk factors in the WA context.

Health service usage measures with significant associations with EHF among different population groups during HW days were compared with those during non-heat wave days. *Via* bivariate analyses, the interactive effects of HW and risk factor were examined to identify vulnerable groups. Risk factors included age group (0–14, 15–59, and 60+ years), gender, SEIFA, ARIA for 2011, and geographical areas [local government areas (LGAs)] sourced from the ABS.

Poisson regression modeling was then used to evaluate the potential association between HW intensity and the number of presentations to EDs and inpatient admissions for heat-related causes. In the models, daily health service usage counts by age group, gender, and SLA were used as a dependent variable and regressed on all potential risk factors described above. The offset variable was daily populations by age group, gender, and SLA. Where an excess of 0 count was identified, zero-inflated Poisson regression was used. Where there was an over-dispersion of counts of health service usage, negative binomial regression was applied.

The interactions between each risk factor and HW intensity were assessed in the regression models. Variables such as public holidays and weekend days were also included in the model to adjust for their confounding effects when assessing the vulnerability of populations to HW.

### Determine Geographical Variations Using Composite Rankings

To compare the health service utilization rates among different geographical regions, both crude rates and age standardized rates (ASRs) were calculated. The 2001 Australian standard population was used for standardization.

Where a health service usage indicator was identified as having a significant association with HW, it was further examined by LGAs. To summarize the overall impact (combined effect) of HW on three significant health service usage indicators (i.e., overall ED attendances, hospitalizations due to heat-related episode, and chronic kidney disease) in different LGAs, the following formula was used to derive a composite score for each LGA.

$$\text{Composite score}=\sum_{j=1}^{3}{\text{DASR}}_{j}\times \frac{{\text{RR}}_{j}}{\sum_{i=1}^{3}{\text{RR}}_{\text{i}}}$$

where DASR*_j_* is the difference of ASRs for a particular health service usage indicator between HW days and non-HW days, and RR*_j_* is the relative risk between HW and non-HW days for that health service usage indicator. Finally, the composite score was divided into five quantiles representing the least, small, median, high, and highest impact of HW for a particular LGA with the highest impact areas being defined as hotspots.

Significant difference was defined as having a *p*-Value <0.05. SAS Enterprise version 5.1 (SAS Institute Inc.) was used for statistical analysis.

Ethics approval for this project was obtained from the WA Department of Health Human Research Ethics Committee. Health service utilization and mortality data are routinely collected by the Department of Health WA. This study was given approval to access and analyze de-identified data to ensure that patient confidentiality was maintained.

## Results

Only results related to ED attendances and hospitalizations due to heat and kidney diseases are presented here, as measures in other datasets were not identified as having significant association with the EHF.

### Association between Risk Factors and Health Usage Measures

Table 1 shows the association between each of the main risk factors and their associations with HW intensity for health service usage measures without adjusting for other risk factors. Only those measures that had a significant association with HW intensity were included. A dose–response relationship between measured health service usage rates and HW intensity was apparent regardless of age group, gender, SEIFA, and ARIA. The more intense the HW, the higher the health service usage rates. For hospitalization, there was also a strong dose–response relationship between age and rates under each HW intensity category. The older the population group, the higher the health service usage rates. However, young age (0–14 years) was more vulnerable to heat than the other two age groups in terms of ED attendance.

**Table 1:** **Crude health service usage rates and 95% CIs by risk factors and HW intensity, November 2006–April 2015, Western Australia**.

Risk factor	Level	HW intensity	Heat-related hospitalization (/10,000,000 per day)	Kidney disease hospitalization (/10,000,000 per day)	Emergency department attendance (/100,000 per day)
Age (years)	60+	No HW^a^	4.55 (4.31–4.79)	114.37 (113.83–114.91)	110.11 (109.96–110.27)
		Low intensity	**11.30 (10.04–12.55)**	**124.79 (122.92–126.66)**	108.83 (108.32–109.33)
		Severe/extreme	**26.26 (21.42–31.10)**	**126.62 (121.87–131.38)**	**115.61 (114.29–116.92)**
	15–59	No HW^a^	1.82 (1.74–1.90)	54.93 (54.74–55.13)	92.61 (92.54–92.68)
		Low intensity	**4.32 (3.91–4.72)**	**60.43 (59.75–61.11)**	92.81 (92.56–93.05)
		Severe/extreme	**7.56 (6.23–8.89)**	**61.23 (59.53–62.92)**	**99.60 (98.97–100.22)**
	0–14	No HW^a^	1.57 (1.43–1.70)	14.17 (13.99–14.35)	125.69 (125.53–125.84)
		Low intensity	2.22 (1.69–2.75)	**15.08 (14.47–15.70)**	**118.41 (117.91–118.91)**
		Severe/extreme	4.24 (2.43–6.06)	**16.50 (14.90–18.10)**	**129.27 (127.98–130.56)**

Gender	Male	No HW^a^	2.94 (2.83–3.05)	53.76 (53.55–53.98)	102.90 (102.82–102.99)
		Low intensity	**7.25 (6.66–7.84)**	**59.49 (58.74–60.25)**	**101.44 (101.16–101.73)**
		Severe/extreme	**12.50 (10.58–14.42)**	**58.84 (56.98–60.70)**	**108.01 (107.29–108.74)**
	Female	No HW^a^	1.53 (1.44–1.61)	60.94 (60.71–61.17)	101.14 (101.05–101.23)
		Low intensity	**2.95 (2.57–3.33)**	**66.21 (65.41–67.02)**	**99.55 (99.27–99.84)**
		Severe/extreme	**7.53 (6.02–9.04)**	**68.46 (66.42–70.50)**	**108.00 (107.26–108.74)**

Socioeconomic index for areas	Most disadvantaged area + Q2	No HW^a^	3.44 (3.22–3.67)	71.61 (71.14–72.07)	198.31 (198.09–198.53)
		Low intensity	**9.30 (8.04–10.55)**	**76.94 (75.33–78.56)**	**190.81 (190.08–191.55)**
		Severe/extreme	**13.08 (9.38–16.77)**	**76.68 (72.68–80.69)**	**213.52 (211.59–215.44)**
	Q3	No HW^a^	2.30 (2.14–2.45)	59.99 (59.63–60.34)	96.09 (95.96–96.22)
		Low intensity	**5.09 (4.32–5.86)**	**65.96 (64.72–67.20)**	**95.26 (94.83–95.69)**
		Severe/extreme	**11.21 (8.35–14.08)**	**66.13 (63.02–69.23)**	**100.72 (99.61–101.82)**
	Least disadvantaged area + Q4	No HW^a^	1.94 (1.86–2.02)	53.13 (52.94–53.32)	81.64 (81.58–81.71)
		Low intensity	**4.21 (3.82–4.61)**	**58.71 (58.05–59.37)**	**82.31 (82.08–82.53)**
		Severe/extreme	**9.03 (7.59–10.47)**	**59.89 (58.23–61.54)**	**87.14 (86.56–87.72)**

Accessibility/remoteness index of Australia	R and VR	No HW^a^	3.99 (3.64–4.34)	69.83 (69.17–70.48)	273.76 (273.38–274.13)
		Low intensity	**14.05 (11.80–16.30)**	69.64 (67.40–71.88)	**258.97 (257.73–260.22)**
		Severe/extreme	**16.81 (11.60–22.02)**	66.69 (62.05–71.32)	**258.88 (256.25–261.52)**
	MA	No HW^a^	3.40 (3.09–3.71)	56.82 (56.25–57.39)	186.01 (185.71–186.30)
		Low intensity	**8.65 (6.77–10.52)**	**61.65 (59.42–63.89)**	**192.51 (191.37–193.65)**
		Severe/extreme	**11.48 (6.57–16.39)**	61.29 (56.22–66.36)	**199.77 (197.13–202.41)**
	A	No HW^a^	2.12 (1.99–2.24)	57.79 (57.51–58.07)	97.58 (97.47–97.68)
		Low intensity	**4.41 (3.84–4.98)**	**63.26 (62.30–64.22)**	**100.37 (100.02–100.72)**
		Severe/extreme	**10.22 (7.98–12.46)**	**65.50 (62.96–68.03)**	**103.22 (102.30–104.14)**
	HA	No HW^a^	1.90 (1.82–1.99)	55.40 (55.18–55.61)	68.96 (68.89–69.03)
		Low intensity	**4.06 (3.64–4.49)**	**61.84 (61.10–62.58)**	**70.83 (70.60–71.06)**
		Severe/extreme	**8.60 (7.04–10.15)**	**62.22 (60.35–64.10)**	**72.06 (71.47–72.64)**

*^a^Reference category for HW intensity; HW, heat wave*.

*Bold numbers denote a higher rate during for the low or severe/extreme HW intensity days compared to non-HW days*.

Males had higher heat-related hospitalization and ED attendance rates than females. However, females had higher rates of hospitalization due to kidney diseases.

There was an apparent dose–response relationship between health service usage rates and SEIFA categories. Overall, the more socially advantaged the population, the lower the rate. The rates during low intensity or severe/extreme HW days were significantly higher than those during non-HW days.

There was also an apparent dose–response relationship between service accessibility and heat-related hospitalization rate. The less remote a population, the lower the health service usage rate.

### Identify Vulnerable Populations through Adjusting for Risk Factors *via* Regression Analyses

Table 2 presents the final regression analysis results showing risk factors and their interaction with HW intensity when examining effects of HW on health service usage measures. Only risk factors with significant interaction with HW intensity were included in the final results.

**Table 2:** **Adjusted rate ratios and 95% CIs of risk and confounding factors for health service usage measures, November 2006 to April 2015, Western Australia**.

Risk factor	Category	Interaction with	Heat-related hospitalization^a^	Kidney disease hospitalization^a^	Emergency department attendance^a^
HW intensity	Severe/extreme		2.120 (1.327–3.387)	1.157 (1.047–1.279)	1.046 (1.037–1.055)
	Low intensity		1.451 (1.223–1.824)	0.988 (0.945–1.032)	1.023 (1.019–1.026)
	No HW^a^				

Age group	60+ years		3.027 (2.737–3.348)	8.041 (7.934–8.151)	1.212 (1.210–1.214)
	15–59 years		1.172 (1.064–1.290)	3.887 (3.836–3.939)	
	0–14 years				1.315 (1.313–1.317)

Gender	Male		1.939 (1.814–2.072)	0.898 (0.893–0.903)	0.992 (0.991–0.993)
	Female				

SEIFA	Most disadvantaged + Q2		1.343 (1.242–1.452)	1.262 (1.252–1.272)	1.546 (1.544–1.548)
	Q3		1.166 (1.085–1.252)	1.119 (1.112–1.127)	1.200 (1.198–1.202)
	Least disadvantaged + Q4				

ARIA	Remote and very remote		2.132 (1.939–2.343)	1.255 (1.242–1.268)	3.269 (3.263–3.275)
	Moderately accessible		1.576 (1.426–1.742)	0.943 (0.933–0.954)	2.243 (2.238–2.247)
	Accessible		1.070 (0.999–1.147)	1.034 (1.027–1.040)	1.322 (1.320–1.324)
	Highly accessible				

Public holiday	Yes		1.102 (0.965–1.258)	1.009 (0.996–1.022)	1.121 (1.118–1.124)
	No				

Month	November		3.444 (2.984–3.975)	1.140 (1.129–1.151)	1.029 (1.027–1.031)
	December		3.636 (3.159–4.184)	1.072 (1.062–1.083)	1.042 (1.039–1.044)
	January		4.572 (3.990–5.238)	1.149 (1.138–1.159)	1.005 (1.003–1.008)
	February		3.135 (2.718–3.616)	1.174 (1.163–1.185)	1.013 (1.011–1.015)
	March		2.236 (1.930–2.589)	1.096 (1.086–1.106)	1.024 (1.022–1.026)
	April				

Year	2015		1.212 (1.070–1.374)	1.529 (1.508–1.549)	1.054 (1.051–1.057)
	2014		1.083 (0.967–1.213)	1.537 (1.519–1.556)	1.067 (1.064–1.070)
	2013		1.111 (0.992–1.244)	1.654 (1.634–1.674)	1.108 (1.105–1.111)
	2012		1.114 (0.997–1.246)	1.626 (1.606–1.646)	1.129 (1.126–1.131)
	2011		0.766 (0.676–0.868)	1.543 (1.523–1.562)	1.124 (1.121–1.127)
	2010		0.773 (0.682–0.876)	1.400 (1.382–1.418)	1.059 (1.056–1.061)
	2009		0.743 (0.652–0.847)	1.208 (1.192–1.224)	1.042 (1.039–1.045)
	2008		0.659 (0.576–0.755)	1.143 (1.127–1.158)	1.021 (1.018–1.024)
	2006		0.579 (0.469–0.715)	1.066 (1.046–1.088)	1.010 (1.006–1.013)
	2007				

Weekend	Weekend		1.042 (0.980–1.108)	0.994 (0.988–1.000)	1.068 (1.067–1.070)
	Weekday				

Age group^a^	60+ years	Severe/extreme	2.142 (1.330–3.450)	0.950 (0.856–1.055)	1.013 (1.000–1.027)
HW intensity					
	60+ years	Low intensity	1.757 (1.326–2.328)	1.023 (0.977–1.070)	0.990 (0.985–0.996)
	60+ years	No HW^a^			
	15–59 years	Severe/extreme	1.521 (0.948–2.440)	0.955 (0.863–1.057)	
	15–59 years	Low intensity	1.666 (1.268–2.189)	1.033 (0.989–1.080)	
	15–59 years	No HW^a^			
	0–14 years	Severe/extreme			0.969 (0.957–0.980)
	0–14 years	Low intensity			0.944 (0.939–0.949)
	0–14 years	No HW^a^			

Gender^a^	Male	Severe/extreme	0.864 (0.665–1.122)	0.969 (0.927–1.012)	0.965 (0.955–0.974)
HW intensity					
	Male	Low intensity	1.277 (1.081–1.508)	1.020 (1.001–1.039)	1.000 (0.996–1.004)
	Male	No HW^a^			
	Female	Severe/extreme			
	Female	Low intensity			
	Female	No HW^a^			

*^a^Under these headings, any cells without RRs and 95% confidence intervals (CIs) are reference categories, and in the brackets are 95% CIs; HW, heat wave*.

First, we observed that there was an apparent dose–response relationship between the HW intensity and health service usage rates. The more intense the HW, the higher the health service usage rates. Those aged 15–59 and 60 years and over were more at risk of heat- or kidney disease-related hospital admissions than those aged 0–15 years. Meanwhile, those aged 0–14, and 60 years and over, had higher chance to attend ED than those aged 15–59 years.

Males had nearly two times higher heat-related hospitalization rates than females. However, females had higher kidney disease-related hospitalization rates and ED attendance rates than males with HW exposure.

There was an apparent dose–response relationship between SEIFA and all three health service usage measures. The more disadvantaged the population, the higher the rate of health service usage.

There was also an apparent dose–response relationship between ARIA and heat-related hospitalization, ED attendance, and kidney disease-related hospitalization rates overall. The less accessible services were, the higher the health impact.

Emergency department attendance rates were higher during pubic holidays and weekend. However, there were no statistically significant differences in admission rates for heat- and kidney disease-related hospital admissions between these two periods. Other variables, such as year and month, were also used to adjust the possible impact of these confounding factors on the three health service utilization rates. Interactions of age and gender with HW effect were also examined. For details of their impact, refer to Table 2.

### Identify Geographical Variation in Population Response to HW

**Figure 1:**
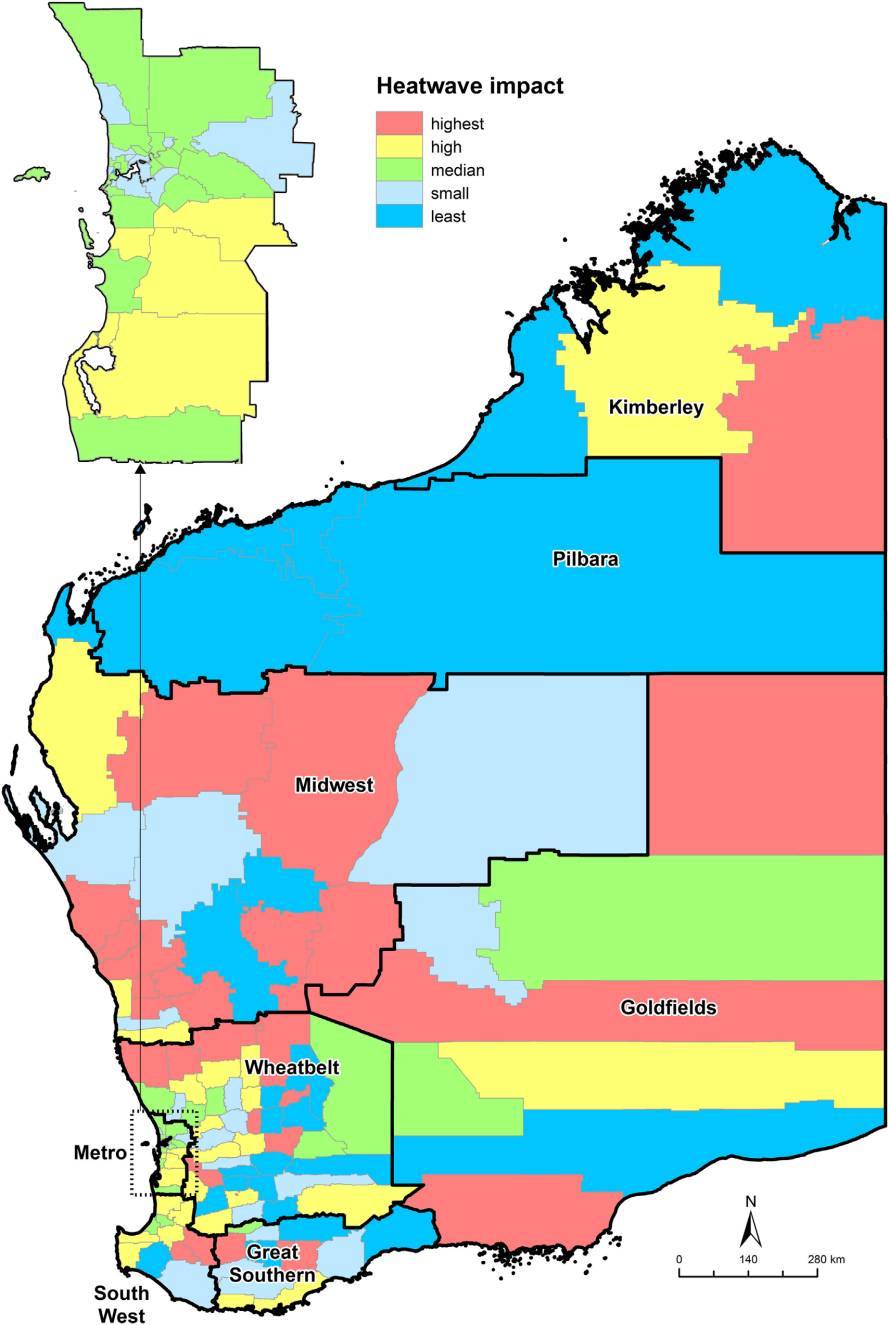
**Heat wave (HW) impact based on composite scores of difference in age standardized rates between HW and non-HW days by local government areas, November 2006 to April 2015, Western Australia**.

Figure 1 shows the composite ranking of the effects of HW by LGA in WA. Only three significant health service usage measures were included in the calculation of the composite ranking. In the populous Perth metropolitan area (as shown in the insert in the map), the overall impacts of HW were between small to high. In the majority of the southern areas, there was a higher impact from HW than the northern areas. However, the highest impact areas were all located in regional and remote areas.

## Discussion

### Sensitivity of Data Sources/Conditions

Outcomes from this study indicated that the heat-related hospitalizations and overall ED presentations were the two most sensitive measures for assessing the impact of HW on health services. Hospital admissions due to kidney diseases were also sensitive. However, overall hospital admissions, hospital admissions due to cardiovascular and respiratory diseases, and all-cause deaths were not sensitive to HW. Similar findings were observed in other studies between HW and ED attendances and hospital admissions due to kidney diseases (18, 19). We also found that the effect of HW on health service indicators examined were not usually immediate and different data sources and conditions had diverse delayed effects of HW. Overall ED attendances and heat-related hospitalizations showed an early effect of HW within 3 and 5 days of a HW event, respectively. This is consistent with some previous studies where lag effects of HW were apparent with a short lag effect for ED attendance data (14, 20, 21). However, kidney disease-related hospital admissions reached their peak rate 25 days after a HW event.

The different lag effect in different data sources is most likely due to varying patient case-mix and structure of the general population involved. In heat-related hospitalizations, only records with heat-related conditions were included. These data sources may potentially fail to identify patients affected by HW but who present to hospital due to exacerbation of pre-existing comorbidities. Although we have excluded elective patients from hospitalization data, in an attempt to identify hospitalizations potentially related to heat exposure, we could not identify an apparent association between all-cause hospitalizations and HW intensity. In ED attendance data, however, all patients were included and potentially heat-related conditions would be included.

Indicators such as heat-related hospitalizations and overall ED attendances can provide responding agencies with insight into the impact of HW on health services. ED datasets are rapidly accessible and could be used for syndromic surveillance. However, hospitalization and mortality data are usually not available for up to 6 months or even longer, which render them unsuited for timely identification of HW-related vulnerable populations and activation of emergency response strategies. Findings from this study reinforce the response strategy of using rapidly accessible ED data to monitor heat-related health impacts during HW events. Therefore, the design of HW service provision must take into account the sensitivity and timeliness of data sources.

### Resilience and Vulnerability to HW

Our study confirmed that age was an important risk factor for HW: people aged 60 years and over were more vulnerable to HW than other age groups and attended health services more frequently, and young people aged 14 and less were more vulnerable to HW for ED services. Gabriel and Endlicher (10) and Tong et al. (11) indicated that the elderly may suffer more due to poor thermoregulation and hormonal changes. Older people with chronic diseases such as cardiovascular and respiratory diseases were particularly vulnerable (22, 23). Previous studies also found that children were at high risk of morbidity and mortality during HWs (8, 9, 14, 24) and children’s inability to lose heat through sweating could cause convulsions and disorientation (17).

Anderson et al. (25) found that there was no significant difference between the vulnerability of males and females during HWs in relation to respiratory hospitalizations. However, our study did identify a significant difference between males and females in heat- and kidney disease-related hospitalizations. Our study observed a higher impact on males than females in heat-related hospitalizations while the study from Rainham and Smoyer-Tomic (26) observed that females had a higher relative risk of mortality than males. This may be due to more men working outside when there is a HW; however, the exact reason warrants further exploration.

Previous studies found that chronic diseases are also risk factors for increased health service utilization among people in extreme heat weather (12–14). People with chronic kidney and cardiovascular conditions are among the most susceptible to heat due to hyperthermia and dehydration. Although our study did not identify a strong association between the rate of hospitalization due to cardiovascular conditions and heat, we did find a strong association between the rate of hospitalization due to chronic kidney diseases and HW intensity, consistent with findings of Nitschke et al. (18) and Williams et al. (19).

Our findings on the main risk factors for HW morbidity were consistent with those identified by Reid et al. (27), which included SES (as indicated by education and poverty), social isolation, and proportion of elderly. The importance of SES in the evaluation of effects of HW was highlighted in several studies of vulnerability to HW (11, 28). Overall, populations with lower SES, poor accessibility to services, and older or younger age groups have higher vulnerability to HWs. The more risk factors in a population, the higher its vulnerability due to the additive feature of the regression models we applied. That means, the contribution of each risk factor would be added up to create a greater health utilization rate. Such vulnerable groups should be the main focus in the development and implementation of HW-related health promotion programs by relevant government and non-government agencies.

The possible joint effects of HW and age or gender were examined in this study and the regression modeling results showed in Table 2. The associations of risk factors and HW intensity were more complicated than expected. For example, the interaction between those aged over 60 years and the intensity of HW on heat-related hospitalization showed a clear dose–response relationship. However, the interaction between the two did not show an apparent dose–response relationship in age group 15–59 years. Instead, the heat-related hospitalization rate increased significantly in age group 15–59 years during low intensity HW exposure. Similarly, patterns were observed on interaction analysis between HW and males on heat-related hospitalization and kidney diseases-related hospitalization. Whether such a pattern was due to the impact from other unexamined risk factors warrants further exploration.

### Regional Differences

This study was able to reinforce some assumptions on HW vulnerability and resilience in regional areas. Depending upon the data sources and conditions, regional responses to HW varied. For example, residents living in far north LGA regions (those with blue colors in Figure 1) with hot dry summer/cool or cold winter climate were least impacted by HWs. However, those living in LGAs with hot dry summer/mild winter climate were more vulnerable to HWs. Physiological acclimatization is likely to be an important factor limiting heat-related health service usage in hot humid or hot dry environment (4), and our study partially confirmed such an observation. However, the sensitivity of data should also be considered for obtaining most suitable health service indicators to explore the effect of HW on health service utilization.

All these regional differences in the study are most likely related to residents’ acclimatization, the region’s SES, accessibility to services, and age/gender and ethnicity distribution of the population as described in other studies (29–31). Which of these factors have played the most important role, and how they interact with each other, warrants further study.

The use of weighted ranking of difference in ASRs between HW and non-HW days by LGA allows us to combine the effects of HW on three data sources/conditions that showed a strong association with effects of HW. Local governments are the main government agencies who would implement the HW strategies. The hotspots identified *via* composite scores will be more reliable than a single health service usage measure in assisting local government agencies to allocate limited resources to those in most need.

### Policy Implications for Emergency Management

As Michelozzi et al. (32) indicated in the consideration of global climate change that the impacts of heat on health will assume greater public health significance in future. The results from this study have significant policy implications for emergency management of HW. This study identified at risk population groups and provided a visual display mapping tool of HW vulnerability and resilience to assist local governments and emergency management regions.

By demonstrating the areas of greatest vulnerability, responding agencies are able to better target prevention and preparedness programs to those most in need. The findings from this study can also be used by local government authorities to better target, engage, and represent the needs of identified at risk groups within their boundaries. The geographical breakdown of HW risk factors will allow responding agencies to better understand and contextualize areas of vulnerability to HWs within their community and appropriately tailor community awareness programs, appropriate risk communication, and HW response plans to the needs of the community.

It is also worth noting that, in the design of the health promotion programs to tackle HWs, identified risk factors should be considered together, so that the programs can be implemented effectively and in an integrated fashion.

### Limitations and Future Directions

The study did not include factors such as air quality measures and their potential interaction with HW intensity measures, as indicated in several studies (33, 34). This study did not include aboriginality as a risk factor, although this is a population group that experiences high rates of chronic kidney and cardiovascular disease (35), and social disadvantage (36). In addition, we did not adjust for the effect of green space on the health outcomes due to unavailability of such data in a vast state such as WA.

Limited diagnostic information in ED data prevented further examination of HW effects on populations with different causes of ill health. Improvements in ED data collection, particularly of diagnostic information, should be considered so that health education messages can effectively target higher risk groups.

Further studies are needed to explore the effects of HW on various disease conditions and on possible mechanisms that explain why populations living in different geographic locations have varied responses to HW. It is also important to conduct evaluation studies to assess the effectiveness of current preventative programs in relation to HW.

## Author Contributions

JX, TS, LJ, GY, and CS designed the study and contributed to draft and revision. JX, LJ, GY, and CS analyzed this work. JN and RF compiled the meteorological data used in the study. JN, RF, AR, and TW provided contribution to drafts and also revisions. All the authors confirmed the last version.

## Conflict of Interest Statement

The authors declare that the research was conducted in the absence of any commercial or financial relationships that could be construed as a potential conflict of interest.
